# Starch-based environment friendly, edible and antimicrobial films reinforced with medicinal plants

**DOI:** 10.3389/fnut.2022.1066337

**Published:** 2023-01-10

**Authors:** Amjad Ali, Abdul Basit, Azhar Hussain, Shehla Sammi, Asif Wali, Gulden Goksen, Ali Muhammad, Furukh Faiz, Monica Trif, Alexandru Rusu, Muhammad Faisal Manzoor

**Affiliations:** ^1^Department of Agriculture and Food Technology, Karakoram International University, Gilgit, Pakistan; ^2^Department of Science and Technology, The University of Agriculture, Peshawar, Pakistan; ^3^Department of Food Science and Technology, The University of Haripur, Harīpur, Pakistan; ^4^Department of Food Technology, Vocational School of Technical Sciences at Mersin Tarsus Organized Industrial Zone, Tarsus University, Mersin, Turkey; ^5^CENCIRA Agrofood Research and Innovation Centre, Cluj-Napoca, Romania; ^6^Biozoon Food Innovations GmbH, Bremerhaven, Germany; ^7^Guangdong Provincial Key Laboratory of Intelligent Food Manufacturing, Foshan University, Foshan, China; ^8^School of Food Science and Engineering, South China University of Technology, Guangzhou, China

**Keywords:** starch, barrier properties, shelf life, medicinal plants, antibacterial properties, tensile properties

## Abstract

In the current study, cornstarch-based antimicrobial and edible films were designed using solution-casting methods. The medicinal plants (*Acontium heterophyllum, Artemisia annua*, and *Thymus serpyllum*) reinforced the gelatinized solution in different concentrations as fillers. The effect of plant extracts on antimicrobial and antioxidant potential, microstructure, barrier, thermal and mechanical properties of cornstarch-based films (SBFs) was investigated using antimicrobial activity, DPPH free radical scavenging values, scanning electron microscopy, X-ray diffraction, water vapor transmission rate, differential scanning calorimetry, and tensile strength. Likewise, it was depicted that the geometric and crystalline structures of medicinal plants’ reinforced films remained the same even after processing. The mechanical tests indicated that the plant extracts effects are associated with reduced elongation, increasing tensile strength, and Young’s modulus. Morphological analysis revealed the generation of uniform and the compact surfaces. However, films with 10% concentration of plant extracts have the lowest water vapor permeability values, and emerged better barrier properties. Moreover, these films showed the significant antioxidant potential and antimicrobial activity.

## 1. Introduction

The therapeutic properties of medicinal plants have been recognized globally; over 50% of recent clinical drugs have been extracted from plant extracts. According to some reports, most of the population (80%) from developing regions use medicinal plants for their primary healthcare (1, 2). Nearly 8,000 plant species with medical values have been reported in South Asia, out of which 1,000 exists in Pakistan. With their indigenous knowledge, such plants were used to treat certain diseases for years (3, 4). However, modern research reported phytochemicals such as alkaloids, glycosides, saponins, resins, and essential oils with other functional compounds such as furanocoumarins and hydroxycoumarins napthoquinones and acylphloroglucinols in these species. Thorough health benefits including cancer, cardiovascular disorders (5–9), ulcer, hemorrhage, diarrhea, and microbial, parasites, and vermifuge treatments with these bioactive compounds, highlighted by numerous researchers (10). In addition, bacterial infections, including gastrointestinal, pneumonia, pulmonary, and skin, are among the common problems treated with antimicrobial medical plants (11).

In addition to other medicinal plants, *Thymus serpyllum* (wild thyme) grows at higher altitudes and possesses various medicinal properties. Some studies proved its anti-rheumatic, antiseptic, antispasmodic, antimicrobial, cardiac, carminative, diuretic diaphoretic, analgesic, carminative, expectorant, and stimulant attributes (12). Likewise, *Artemisia annua* L. (sweet wormwood) is also a medicinal plant, widely available in Europe, Africa, China, and Pakistan (13, 14). Due to rich bioactive compounds, the plants act as anti-rheumatic anthelmintic, antispasmodic, and antibacterial natural sources. Additionally, treatment of malaria, hepatitis, cancer, inflammation, and menstrual associated issues are also treated with the plants. Moreover, *Aconitum heterophyllum* is also a Himalayan medicinal specie, beneficial against, fever, diarrhea, digestive problems, nervous system, rheumatism issues and reported to possess antifungal, antiviral, and immune-stimulant attributes (15). Furthermore, in the traditional medicine of China, India, and Pakistan, the plant is beneficial for treating sciatica and rheumatic pain and can also be used against body lice (16, 17).

In recent years, numerous antimicrobial packaging systems have been introduced with different polymer matrices (18–20). However, these films possess certain limitations such as toxicological effects, costly environmental issues, poor mechanical attributes, high water affinity, and others (21, 22). On the other hand, using antimicrobial materials from natural sources is among emerging research topics in preserving food from microbial contaminations and developing anti-microbial food packaging with numerous advantages (23, 24). For example, reinforcement of SBFs with natural fillers having biocompatible and biodegradable valuable nature in medicine, drug release, packaging, and agricultural research (25, 26). The natural fillers can act as a reinforcing source and enhance the matrix’s mechanical and barrier characteristics by delivering matrix tension to the fillers (27).

Despite the efforts, no attempt has been made to incorporate these medicinal plants as fillers in SBFs to prepare films with unique characteristics. Therefore, in the current work, the effect of *Acontium heterophyllum, Artemisia annua*, and *Thymus serpyllum* medicinal plant from Pakistan’s northern part (Gilgit region), as novel and iinovative approach was evaluated on the environmental-friendliness, thermo-mechanical, and antibacterial attributes of the SBFs. Since all the components employed were derived from food sources; therefore, it is anticipated that the material would possess great potential as antimicrobial packaging for food in future research.

## 2. Materials and methods

### 2.1. Materials

Hydroxypropyl high-amylose cornstarch (amylose content 80%) was purchased from Penford, Australia. Fresh medicinal plants (*Acontium heterophyllum, Artemisia annua*, and *Thymus serpyllum*) were collected from the upper pastures of Bagrote valley located at Central Karakoram National Park Gilgit Baltistan, Pakistan. After identification, washing, and cleaning with distilled water, the plants were frozen (at −85°C) in the presence of liquid nitrogen. Then the sample was dried with a freeze dryer (FDU-1200 EYELA) at a vacuum pressure and condenser temperature of 7 m Torr and −88°C, respectively, for 24 h. The freeze-dried plants were finally granulated into a fine powder, followed by sieving with a 180 mesh size.

### 2.2. Film preparation

The following solution casting procedure synthesized all the films. Briefly, a corn starch solution (8%, w/w) was prepared in a conical flask, and then 20% polyethylene glycol was injected on a dry weight basis of starch. Further, the solution was pre-mixed and then heated to 99°C (maintained for 1 h) with continued agitation. The solution was further cooled at 55°C, and fillers (0, 5, 10, and 15%) were mixed into the gelatinized suspension and agitated for 40 min. Moreover, the mixed suspensions were poured into Petri dishes and were coded according to the medicinal plant (see Table 1). The Films were then dried for 8 h in an oven (at 37 ± 1°C) to get constant weight.

**TABLE 1:** Sample codes of films reinforced with different medicinal plants.

Sr. no	Code	Medicinal plant	Code	Filler (%)	Weight of each component (g)				Final volume
					PEG	Starch	Filler	Water	
1	PSF	Corn starch	PSF	0	1.6	8	0	90.4	100
2	AH1	*Accountium heterophyllum*	AH1	5	1.6	8	0.4	90	100
3	AH2	*Accountium heterophyllum*	AH2	10	1.6	8	0.8	89.6	100
4	AH3	*Accountium heterophyllum*	AH3	15	1.6	8	1.2	89.2	100
5	AA1	*Artemisia annua*	AA1	5	1.6	8	0.4	90	100
6	AA2	*Artemisia annua*	AA2	10	1.6	8	0.8	89.6	100
7	AA3	*Artemisia annua*	AA3	15	1.6	8	1.2	89.2	100
8	TS1	*Thymus serpyllum*	TS1	5	1.6	8	0.4	90	100
9	TS2	*Artemisia annua*	TS2	10	1.6	8	0.8	89.6	100
10	TS3	*Artemisia annua*	TS3	15	1.6	8	1.2	89.2	100

### 2.3. Characterizations of films

#### 2.3.1. Transparency measurement

A UV (WFZ UV-3802) spectrum was employed to estimate the prepared films’ transparency. The samples were first placed in a 10 × 10 mm square container for the calculations. A wavelength of 315 nm corresponds to transparency in the research. The transparency of various films was also observed at 206 nm, divided by thickness and displayed as %/mm.

#### 2.3.2. Water vapor transmission rate (WVTR)

Water vapor transmission rate (WVTR) of samples was evaluated with a thermos hygrometer using deionized water following ASTM E96/E96M-14 guidelines. The films were first placed in a permeation cell sealed over a circular opening of 2.82 cm^2^, then stored in a desiccator (at 25 °C) to maintain a 75% relative humidity (RH). Water vapor permeability was calculated as weight gain on the permeation cell. Other researchers also employed this methodology (28, 29).

#### 2.3.3. Scanning electronic microscope (SEM)

For observing microstructural surface structure with scanning electronic microscope (SEM) (PHENOM Pro) technique, the films were adjusted on copper stubs and then coated with gold. Finally, the samples were estimated by setting an accelerating voltage of 5 kV.

#### 2.3.4. Tensile testing

The ASTM D5938-96 standard Instron tensile testing apparatus (5,565) was used to test tensile strength. Tensile strength, elongation at break and modulus was estimated at a crosshead speed of 5 mm/min. Seven specimens were evaluated for each film and mean values were obtained.

#### 2.3.5. X-ray diffraction (XRD)

The X-ray patterns of corn starch-based films reinforced with three different medicinal plants as a filler were tested with an X-ray diffractometer with Cu K radiation at 40 kV and 30 mA voltage. The samples were scanned between 2θ = 3 and 40°.

#### 2.3.6. Differential scanning calorimetry (DSC)

Differential scanning calorimetry (DSC) test was performed with a Q1000 DSC system (TA instruments, USA). Different film pieces (10 mg) were sealed in a standard aluminum pan followed by heating at a rate of 5°C/min from −25 to 550°C under a nitrogen atmosphere.

### 2.4. Antimicrobial activity of films

The SBFs’ anti-microbial activity was ascertained with the disk diffusion technique proposed by Zaiden et al. (30). The current protocol followed a modified agar disk diffusion approach against the microorganisms: *Staphylococcus aureus*, and *Salmonella*. Firstly, 100 μL of indicator microorganisms containing a bacterial load was put on soft agar and poured over a nutrient agar plate to prepare a layer of indicator microorganisms. Further, the plates were incubated at 37°C (overnight) for solidification. The prepared corn starch-based films of 6 mm diameter were put onto the surface of a nutrient agar plate, followed by incubation at 37*^o^*C for 24 h. The inhibitory level of the films was presented as zone inhibition in diameter (mm) around the disk. All the films were evaluated in triplicate, and the average mean of the inhibition zone inhibition was determined.

### 2.5. DPPH radical scavenging activity

The % DPPH radical scavenging activity of the corn starch-based films was measured by following the method proposed by Brand-Williams et al. (31). To calculate the antioxidant properties of prepared films, 5 g of each film was homogenized and then extracted with 50 mL of methanol for 6 h. Further, 0.1 mL of methanol extract was taken in a flask, and 4.9 mL of DPPH solution was added and kept in the darkroom for incubation (30 min). After incubation, the absorbance was recorded at 715 nm with a UV spectrophotometer. The antioxidant activity was calculated with the following expression:


Antioxidantactivity(%)

$$=\frac{\mathrm{Absorbance}\,\mathrm{blank}-\mathrm{Absorbance}\,\mathrm{with}\,\mathrm{sample}}{\mathrm{Absorbance}\,\mathrm{blank}}\times 100$$

### 2.6. Solubility in water

Film solubility is the % calculation of total soluble matter (TSM) in the film and is solubilized in distilled water (32). The films’ initial weight (0.50 mg) was calculated and dried in an oven at 70 ± 5°C for 24 h. After drying, the initial weight was calculated, and the dried samples were immersed in 50 g of distilled water for 6 h (at 25°C). After 6 h, the insoluble films were again dried at 70 ± 5°C until a constant weight was attained. The %TSM was calculated by using the following formula:


%⁢T⁢S⁢M

$$=\frac{I\,n\,i\,t\,i\,a\,l\,w\,e\,i\,g\,h\,t\,o\,f\,t\,h\,e\,f\,i\,l\,m-F\,i\,n\,a\,l\,w\,e\,i\,g\,h\,t\,o\,f\,t\,h\,e\,f\,i\,l\,m}{I\,n\,i\,t\,i\,a\,l\,w\,e\,i\,g\,h\,t\,o\,f\,t\,h\,e\,f\,i\,l\,m}\times 100$$

### 2.7. Statistical analysis

The statistical analysis was performed with STATISTICS 8.1 software. The complete randomized design (CRD), analysis of variance (ANOVA), and LSD comparison was performed (33). To detect the difference between the film properties (*p* ≤ 0.05).

## 3. Results and discussion

### 3.1. Physical properties

The effect of the medicinal plants on the thickness, transparency, solubility, moisture, and WVTR of the corn starch film is shown in Table 2. The thickness was controlled by adding the same weight (25 mL) of suspension into the petri dish. However, a slight increase in the film thickness was increased with the increase of filler content in all medicinal plant-reinforced films. The moisture content of the pure corn starch and all reinforced film were the same. It was noticed that adding fillers to the corn starch-based film decreased the transparency level of the films due to the proper dispersion of the filler in the starch matrix. The film-barrier properties against UV radiation of a film is e used to measure at 206 nm to check the transparency level. The declined pattern of opacity was noticed in all e all reinforced films, from 73 to 53% in AA3 reinforced film. The finding indicated that adding medicinal plants to starch-based films improved their UV radiation protection properties (Table 2).

**TABLE 2:** Effect of starch based films reinforced with different medicinal plants on physical properties of films.

Sample	Physical properties				
	Thickness (mm)	Opacity (%/mm)	Solubility (%)	WVTR (g/m^2^.24 h)	Moisture content (%)
Corn starch	0.11 ± 0.06e	73 ± 3a	37.20 ± 3a	1278.00 ± 32a	15.98
Accountium heterophyllum	0.11 ± 0.50e	62 ± 4e	31.51 ± 2e	1220.50 ± 25d	15.80
Accountium heterophyllum	0.13 ± 0.04c	65 ± 5ef	25.88 ± 1h	1186.60 ± 37ef	15.88
Accountium heterophyllum	0.16 ± 0.06a	55 ± 5d	30.600 ± 1f	1250.50 ± 15c	15.87
Artemisia annua	0.12 ± 0.05d	56 ± 4e	33.51 ± 2d	1150.67 ± 15e	15.90
Artemisia annua	0.114 ± 0.06b	68 ± 7b	26.21 ± 3g	1141.25 ± 10g	15.91
Artemisia annua	0.16 ± 0.04a	53 ± 4g	36.09 ± 0.10b	1285.63 ± 32c	15.89
Thymus serpyllum	0.13 ± 0.05c	56 ± 4e	33.02 ± 0.10d	1170.50 ± 21f	15.82
Thymus serpyllum	0.14 ± 0.05b	68 ± 7c	34.07 ± 0.10c	1191.10 ± 27e	15.95
Thymus serpyllum	0.16 ± 0.05a	70 ± 5b	37.07 ± 0.10a	1273.11 ± 11b	15.45

Mean values in the same column with different letters are significantly different (*p* ≤ 0.05). The different letters represent the statistical application that shows data significantly different from each others.

The water solubility rate greatly affected the film’s water vapor resistance quality. Table 2 represents the water solubility results of corn starch-based reinforced film (*p* ≤ 0.05). All the films were statistically different from each other except TS3. It can be seen that the water solubility rate of all films was reduced compared to pure corn starch film. The solubility rate decreased from 37.20 to 25.88%. It was due to the interaction between the starch matrix and the functional compounds in medicinal plants (34).

Table 2 also presents the effect of different medicinal plants on the WVTR of the corn starch film reinforced with different medicinal plants. It was observed that all three medicinal plants having different concentrations reduced the WVTR. Generally, the WVTR of the pure starch film was higher, and it was also noticed that the films with a higher concentration of filler (15%) showed higher WVTR than the lower amount. The highest WVTR (1278 ± 32 g/m^2^ 24 h) was observed in the pure starch film, while the lowest WVTR (1141.25 ± 10 g/m^2^ 24 h) was demonstrated by *Artemisia annua* (10%). A similar study of the role and function of cellulose and chitin nanofibers (CNF) and nanocrystals (NC) have been reported because the homogeneous scattering is essential for barrier properties, and the higher amount of filler also results in the agglomeration of filler particle and weak the WVTR and gas barrier properties (35). The decrease in water vapor transmission rate with adding the medicinal plant powder as a filler was also ascribed to better barrier properties. A stronger interfacial adhesion between starch and matrix was formed, reducing the longer diffusive path and also helping to minimize the penetration of the molecular permeability (36). The network developed between cellulose and starch matrix due to the hydrogen bonding banned the formation of free spaces where water molecules can enter. Our results agree with the previous studies published to compare fillers with different morphologies and their role in starch-based films (37, 38).

### 3.2. DPPH radical scavenging activity (%)

The main characteristic of a packaging film is its antioxidant activity because it protects the food commodity from microbial spoilage and extends the product’s shelf life (39). Table 3 shows the effect of DPPH radical scavenging activity (%) of medicinal plants reinforced starch-based film. All the reinforced films were statistically different (*p* ≤ 0.05). The percent antioxidant activity of starch film reinforced by medicinal plants as a filler enhanced from 0.00 to 69% in 15% *Thymus serpyllum* medicinal plant reinforced film. It was noticed that the higher amount of filler showed higher antioxidant activity in all three different medicinal plant reinforced films as compared to the low amount. A similar increase in antioxidant activity has been reported in the chitosan-starch-based film when incorporated with mango leaf and honeysuckle flower extracts (40, 41). The increased antioxidant activity was due to functional groups in Chinese chive (Allium *tuberosum*) root extract (CRE). The increase in antioxidant activity was due to the higher total phenolic in chitosan-starch-based film reinforced with CRE. Similar trends of improved antioxidant activity were ascribed by the chitosan-starch-based film reinforced by β-carotene and starch NC (42).

**TABLE 3:** Effect of starch-based films reinforced with different medicinal plants on DPPH radical scavenging activity and anti-microbial activity.

S. no	Samples	DPPH radical scavenging activity (%)	Diameter of inhibition zone (mm)	
			*S. aureus*	*Salmonella*
1	Corn starch	0.00 ± 0i	0.0 ± 0g	0.00 ± 0f
2	*Accountium heterophyllum*	39.32 ± 5h	10.0 ± 1.0f	8.0 ± 0.5e
3	*Accountium heterophyllum*	48.66 ± 2g	11.0 ± 0.7de	9.0 ± 0.8d
4	*Accountium heterophyllum*	57.50 ± 4d	13.0 ± 1.8b	12.0 ± 1.0b
5	*Artemisia annua*	49.32 ± 3f	11.0 ± 1.0e	9.0 ± 0.5d
6	*Artemisia annua*	52.43 ± 3e	12.8 ± 1.1c	11.0 ± 0.5c
7	*Artemisia annua*	62.00 ± 5b	14.5 ± 2.0ab	12.0 ± 0.8b
8	*Thymus serpyllum*	68.00 ± 6c	13.0 ± 1.8b	9.0 ± 1.0d
9	*Thymus serpyllum*	62.67 ± 5b	12.5 ± 1.3d	12.0 ± 0.9b
10	*Thymus serpyllum*	69.66 ± 8a	15.0 ± 1.8a	13.0 ± 0.4a

Mean values in the same column with different letters are significantly different (*p* ≤ 0.05). The different letters represent the statistical application that shows data significantly different from each others.

### 3.3. Antimicrobial activity

The packaging material used for packing should possess good antimicrobial activity, protecting the food commodity from bacterial contamination (43). Two different strains, *Staphylococcus aureus* and *Salmonella* were tested to evaluate the antibacterial activity of the medicinal plants’ reinforced films. As shown in Table 3, the starch-based films filled with different medicinal plants indicated good inhibition zones (*p* ≤ 0.05) against both microorganisms. The rate of zone inhibition (diameter) was increased with the increasing amount of medicinal plants in starch-based films compared to the control film. All three medicinal plants presented the highest inhibitory effects (*Accountium hetrophyllum, Artemisia annua*, and *Thymus serpyllum*), having 15% reinforced filler against both microorganisms. The Starch-based films filled with medicinal plants were more effective against Gram-negative and Gram-positive bacteria. Functional ingredients present in medicinal plants, such as total phenolics, Sulfur containing compounds, flavonoids, and allicin, are responsible for the antimicrobial properties (44). The mechanism of pomegranate peel particles against microorganisms can be ascribed to the phenolic toxicity that affects the sulfhydryl groups of proteins present in microorganisms (45, 38). The results agree with previous studies of pomegranate peel against Gram-positive and Gram-negative bacteria. Antibacterial properties of phenolic compounds result in physiological changes in a cell membrane that lead to cell death in bacteria (46).

### 3.4. Tensile properties

Table 4 shows the effect of three different medicinal plants on the tensile properties of the starch films. It was observed that medicinal plants increased the modulus from 387.6 to 483 MPa and tensile strength from 16.65 to 19.12 MPa and decreased the elongation at break from 22.77 to 15.05%. The medicinal plant *Accountium hetrophyllum* (5%) reinforced film detected the best values, followed by *Artemisia annua* (10%) and *Thymus serpyllum.* It was observed that the films with smaller particles display good mechanical properties, which is expected since smaller particles distribute evenly. Filler surface polarity, composition, roughness, adhesion, and wettability are essential in improving reinforced film properties. That affects the barrier and the mechanical properties. It displayed that the medicinal plant powder acted as a natural filler and improved the tensile properties of the film. The contrivance of reinforcement can be clarified by good compatibility between filler and starch. Our previous findings are in line with the recent findings of improvement in mechanical properties (38, 47).

**TABLE 4:** Effect of starch-based films reinforced with different medicinal plants and tensile properties of films.

S. no	Samples	Tensile properties		
		Young’s modulus (MPa)	TS (MPa)	Elongation (%)
1	Corn starch	387.6 ± 12.0e	16.65 ± 1.2d	22.77 ± 3.3a
2	*Accountium heterophyllum*	483.7 ± 14.8a	19.12 ± 0.5a	15.05 ± 1.2d
3	*Accountium heterophyllum*	465.8 ± 13.3c	18.35 ± 1.8ab	16.98 ± 0.8b
4	*Accountium heterophyllum*	454.9 ± 10.8d	17.73 ± 0.8c	16.04 ± 1.4b
5	*Artemisia annua*	468.5 ± 16.4b	17.93 ± 1.1b	15.55 ± 0.8d
6	*Artemisia annua*	476.9 ± 12.1ab	18.86 ± 1.6a	16.00 ± 1.3c
7	*Artemisia annua*	461.4 ± 10.6c	17.98 ± 1.2b	17.19 ± 1.3b
8	*Thymus serpyllum*	470.8 ± 12.7b	18.86 ± 1.6a	16.98 ± 0.8b
9	*Thymus serpyllum*	468.1 ± 11.9b	18.10 ± 0.9a	16.00 ± 0.8ab
10	*Thymus serpyllum*	465.8 ± 13.3c	16.73 ± 0.8d	17.14 ± 1.4b

Mean values in the same column with different letters are significantly different (*p* ≤ 0.05). The different letters represent the statistical application that shows data significantly different from each others.

### 3.5. Differential scanning calorimeter (DSC)

Table 5 shows the results of starch-based films reinforced with different medicinal plants. All the prepared films were heated from −25 to 180°C with a heating rate of 10 C/min. It can be observed from the endothermic curves of starch-based films. The increase in endothermic heat flow was observed in medicinal plant powder-filled films. It was also observed that the endothermic heat flow increased gradually with the increase in medicinal plant powder contents. Understandably, the increase in filler level increased the crystallinity and filler and starch matrix interaction. It reduced the mobility of the amorphous region due to the cross-links made by crystallization (48). The starch-based films reinforced with medicinal plants exhibited a declining peak temperature trend and higher thermal stability.

**TABLE 5:** Effects of different medicinal plants reinforced films on endothermic curves of starch-based films.

Sample	To (*^o^*C)	Tp (*^o^*C)	Tc (*^o^*C)	△H (J/g)
Corn starch	33.59 ± 0.57bc	83.60 ± 0.08e	131.09 ± 0.15a	177.57 ± 2.69d
*Accountium heterophyllum*	35.29 ± 0.68a	84.64 ± 0.12d	130.91 ± 0.02ab	203.20 ± 5.20c
*Accountium heterophyllum*	34.66 ± 0.20abc	85.61 ± 0.04c	129.35 ± 0.04e	208.20 ± 4.23bc
*Accountium heterophyllum*	33.49 ± 0.35cd	91.26 ± 1.74a	129.80 ± 0.02d	237.84 ± 9.83a
*Artemisia annua*	34.20 ± 0.36abc	82.98 ± 0.71f	130.76 ± 0.03b	200.93 ± 5.92c
*Artemisia annua*	34.52 ± 0.66abc	84.26 ± 0.50d	130.23 ± 0.06c	196.94 ± 5.21c
*Artemisia annua*	35.02 ± 0.02abc	87.22 ± 0.21b	128.57 ± 0.36f	216.08 ± 12.88b
*Thymus serpyllum*	33.62 ± 1.50c	84.54 ± 0.22d	129.50 ± 0.22e	200.00 ± 3.22c
*Thymus serpyllum*	34.60 ± 0.22abc	86.13 ± 1.00bc	130.70 ± 0.30b	203.90 ± 2.20c
*Thymus serpyllum*	33.45 ± 1.12cd	88.05 ± 0.10b	128.00 ± 0.30f	210.80 ± 10.98b

To, onset temperature; Tp, peak temperatures; Tc, conclusion temperatures; △H, enthalpy of gelatinization. The different letters represent the statistical application that shows data significantly different from each others.

The increased thermal stability of starch/polyvinyl alcohol films with the addition of nano-silicon dioxide has been reported (49). The result of an improved melting temperature of the starch-based film agrees with (50), who reported that the increase in melting temperature of the composites reinforced with starch NC and waterborne polyurethane matrix.

### 3.6. SEM and XRD observations

**FIGURE 1:**
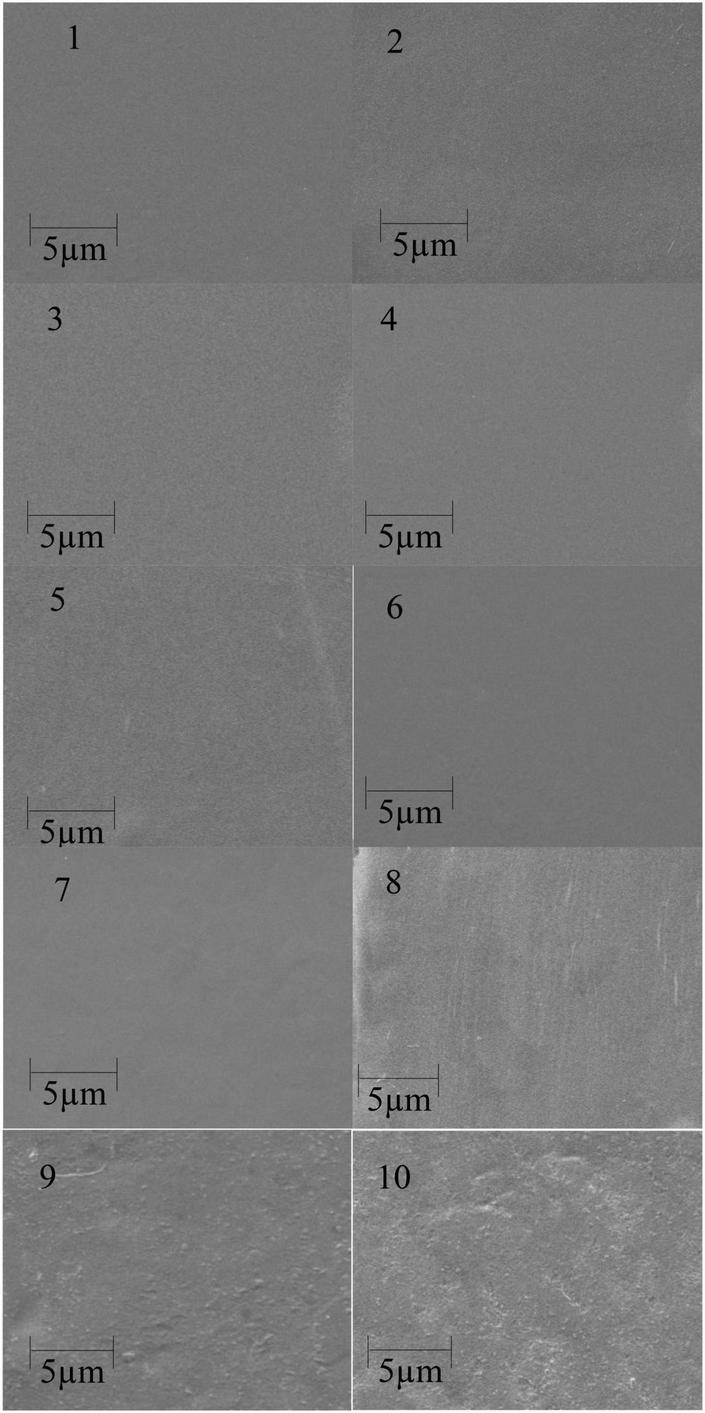
Scanning electronic microscope (SEM) surface images of pure corn starch (1) and the films containing 5% (2–4), 10% (5–7), and 15% (8–10) *Accountium heterophyllum, Artemisia annua*, and *Thymus serpyllum* medicinal plants.

Figure 1 shows the SEM images of corn starch-based films filled with medicinal plants. It is seen that the starch film (1) has a reasonably smooth surface. All three medicinal plants’ reinforced film (5 and 10%) could be seen on the film surface after drying the starch film. The films reinforced with 5% medicinal plant powder were smooth compared to the 10% reinforced films in all three medicinal plants. The rough surface of the films shows good interaction between filler and corn starch matrix. This observation can explain the improved mechanical properties (WVTR) and UV radiations (51, 52).

**FIGURE 2:**
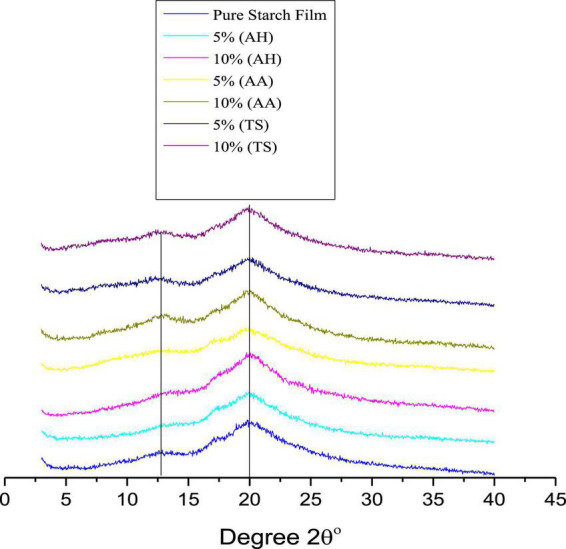
X-ray diffraction (XRD) of pure corn starch (1) and films reinforced with three different medicinal [*Accountium heterophyllum (AH), Artemisia annua (AA)*, and *Thymus serpyllum (TS)*] medicinal plants.

The results indicated that the medicinal plant powder remained stable during film preparation and improved the film’s peak intensity in XRD studies, as shown in Figure 2. The XRD results also support this conclusion (Figure 2). The films showed peaks at 2θ° = 22.20°, associated with starch crystallinity. It was observed that the peak intensity was improved after adding medicinal plant powder as a filler. These results can be used to clarify the reinforcement mechanism in corn starch-based films. Furthermore, the improved mechanical properties can be benefited by developing a rigid network between filler and starch matrix that helps transfer the stress from the matrix to the fillers.

## 4. Conclusion

In the current attempt, corn starch-based antimicrobial films were prepared by reinforcing *Acontium heterophyllum, Artemisia annua*, and *Thymus serpyllum* (medicinal plants) as a filler using a solution-casting approach. Findings determined higher DPPH radical scavenging activity and antimicrobial activity for reinforced films than the pure corn starch film. Moreover, the mechanical properties (Tensile strength and Young’s modulus) of the SBFs significantly improved after reinforcing medicinal plant powder. These results depicted more vital films after reinforcement of natural fillers than without fillers. Furthermore, the barrier properties (WVTR) of the corn starch films were also improved by adding medicinal plant powder. Films reinforced with smaller amounts (5 and 10%) in all three medicinal plants displayed better mechanical properties than films reinforced with significant particle filler content. SEM and XRD observations showed that the compatibility between corn starch and filler was quite good, which was expected. The XRD observation represented that medicinal plant geometry and crystalline structures remained the same in the films after processing, which explained the reinforcement mechanism. The DSC indicated that the prepared samples were more thermally stable than pure corn starch films. Since all the components were from natural food resources, the materials are fully biodegradable, safe for food packaging, and can also be suitable for producing sustainable edible films and medicinal capsules.

## Data availability statement

The raw data supporting the conclusions of this article will be made available by the authors, without undue reservation.

## Author contributions

AA, AB, and AH: conceptualization. AA, SS, AW, and AM: analysis. FF, GG, and MM: methodology. AA, MT, and AR: software. AA and MM: writing—original draft preparation. GG, MM, MT, and AR: writing—review and editing. AA: supervision. All authors contributed to the article and approved the submitted version.
